# Efficiency of antenatal care and childbirth services in selected primary health care facilities in rural Tanzania: a cross-sectional study

**DOI:** 10.1186/1472-6963-14-96

**Published:** 2014-02-28

**Authors:** Happiness P Saronga, Els Duysburgh, Siriel Massawe, Maxwell A Dalaba, Germain Savadogo, Pencho Tonchev, Hengjin Dong, Rainer Sauerborn, Svetla Loukanova

**Affiliations:** 1 Muhimbili University of Health and Allied Sciences (MUHAS), Dar es Salaam, Tanzania; 2 International Center for Reproductive Health (ICRH), Ghent University, Ghent, Belgium; 3 Navrongo Health Research Center, Navrongo, Ghana; 4 Centre de Recherche en Santé de Nouna (CRSN), Nouna, Burkina Faso; 5 Clinic of Surgery, University Hospital “Dr. G.Stranski”, Pleven 5800, Bulgaria; 6 Centre for Health Policy Studies, School of Public Health, Zhejiang University, Hangzhou, China; 7 Department of Public Health, University of Heidelberg, Heidelberg, Germany

**Keywords:** Antenatal care, Childbirth, Cost, Unit cost, Quality, Efficiency, Rural area, Tanzania, Health care provider

## Abstract

**Background:**

Cost studies are paramount for demonstrating how resources have been spent and identifying opportunities for more efficient use of resources. The main objective of this study was to assess the actual dimension and distribution of the costs of providing antenatal care (ANC) and childbirth services in selected rural primary health care facilities in Tanzania. In addition, the study analyzed determining factors of service provision efficiency in order to inform health policy and planning.

**Methods:**

This was a retrospective quantitative cross-sectional study conducted in 11 health centers and dispensaries in Lindi and Mtwara rural districts. Cost analysis was carried out using step down cost accounting technique. Unit costs reflected efficiency of service provision. Multivariate regression analysis on the drivers of observed relative efficiency in service provision between the study facilities was conducted. Reported personnel workload was also described.

**Results:**

The health facilities spent on average 7 USD per capita in 2009. As expected, fewer resources were spent for service provision at dispensaries than at health centers. Personnel costs contributed a high approximate 44% to total costs. ANC and childbirth consumed approximately 11% and 12% of total costs; and 8% and 10% of reported service provision time respectively. On average, unit costs were rather high, 16 USD per ANC visit and 79.4 USD per childbirth. The unit costs showed variation in relative efficiency in providing the services between the health facilities. The results showed that efficiency in ANC depended on the number of staff, structural quality of care, process quality of care and perceived quality of care. Population-staff ratio and structural quality of basic emergency obstetric care services highly influenced childbirth efficiency.

**Conclusions:**

Differences in the efficiency of service provision present an opportunity for efficiency improvement. Taking into consideration client heterogeneity, quality improvements are possible and necessary. This will stimulate utilization of ANC and childbirth services in resource-constrained health facilities. Efficiency analyses through simple techniques such as measurement of unit costs should be made standard in health care provision, health managers can then use the performance results to gauge progress and reward efficiency through performance based incentives.

## Background

Maternal and newborn mortality and morbidity remain among the top global health challenges despite various efforts and multitude of resources directed to this field overtime. The high mortality and morbidity rates, especially in developing countries, necessitate prioritization of greater efficiency as part of efforts towards their reduction. The majority of the 40 countries with the highest maternal mortality in the world are in sub-Saharan Africa [1]. In developing countries, a woman is 15 times more likely to die from, mostly preventable or treatable, pregnancy or childbirth related complications than in the rest of the world [1]. Tanzania is one of the seven countries in the world accounting for 3% to 5% of global maternal deaths reported in 2010 [2].

Although the maternal mortality ratio (MMR) has been slowly declining in Tanzania over the past years, it is still estimated to be at a staggering 454 deaths per 100,000 live births [3]. The neonatal mortality rate per 1000 live births is 26 and the lifetime risk of maternal death in 2010 was 1 per 38 [4,2]. Infant mortality rate (IMR) has been reduced over the past ten years, and in 2011 it was 45 deaths per 1,000 live births [5]; this rate is nevertheless far above that of high income countries. Neonatal mortality accounts for 47% of IMR in Tanzania. The government continuously directs efforts at reducing these challenges, as is demonstrated by various policies, goals, reforms and strategic plans focused on quality, access, equity and efficiency in the provision of MNC services [6].

Faced with resource scarcity coupled with competing development priorities, Tanzania has strived over the years to increase its budget to health sector in its bid to increase both the quality and quantity of essential health services, including MNC services, for its population. In general, money for health in Tanzania comes from public (comprising of taxes and foreign assistance) and private (insurance and user fees) sources. MNC services are free of charge at all levels of health care in public health facilities to encourage utilization. The health spending per capita was at around 27 USD in 2009, including MNC [7]. Comparatively this expenditure is low. Worldwide per capita expenditure ranges from 11 USD in Eritrea to 8262 USD in Luxembourg per year [1]. This reflects, among others, constrained MNC services provision in Tanzania.

Despite the meagerness of resources allocated to health sector, including MNC, it remains paramount to have knowledge of how exactly these scarce resources are spent. Cost studies help to demonstrate how resources have been spent, what resources are needed to provide specific services, areas where costs could be reduced and where output could be increased and thus improve efficiency in the provision of health services [8-10]. There is still only a limited amount of information of this type regarding the specific provision of ANC and childbirth services. The few studies conducted in Tanzania have looked at MNC costs in urban settings [8], at faith based hospitals [11] or taken the consumer/household perspective on costs of MNC [12]. Other studies on MNC were conducted in other developing countries [9,10,13-19].

This study took a health care provider perspective and accounted the actual resources used in the provision of ANC and childbirth services in eleven rural health facilities of two resource-poor districts in Tanzania. The unit costs reflected efficiency in the provision of MNC services. To understand the differences in service provision efficiency among the health facilities, regression analysis of provider and consumer side factors against unit costs was undertaken. We hope the information will help district health management teams to plan efficient resource use.

## Methods

This study is part of an ongoing research project that was granted ethical approval from the Muhimbili University of Health and Allied Sciences (MUHAS) ethics committee (reference number MU/RP/AEC/Vol.XIII/1) and the Faculty of Medicine of the University of Heidelberg, Germany, ethics commission (reference number S173/2008). Likewise, written informed consent was sought from all participants.

### Study area

**Figure 1:**
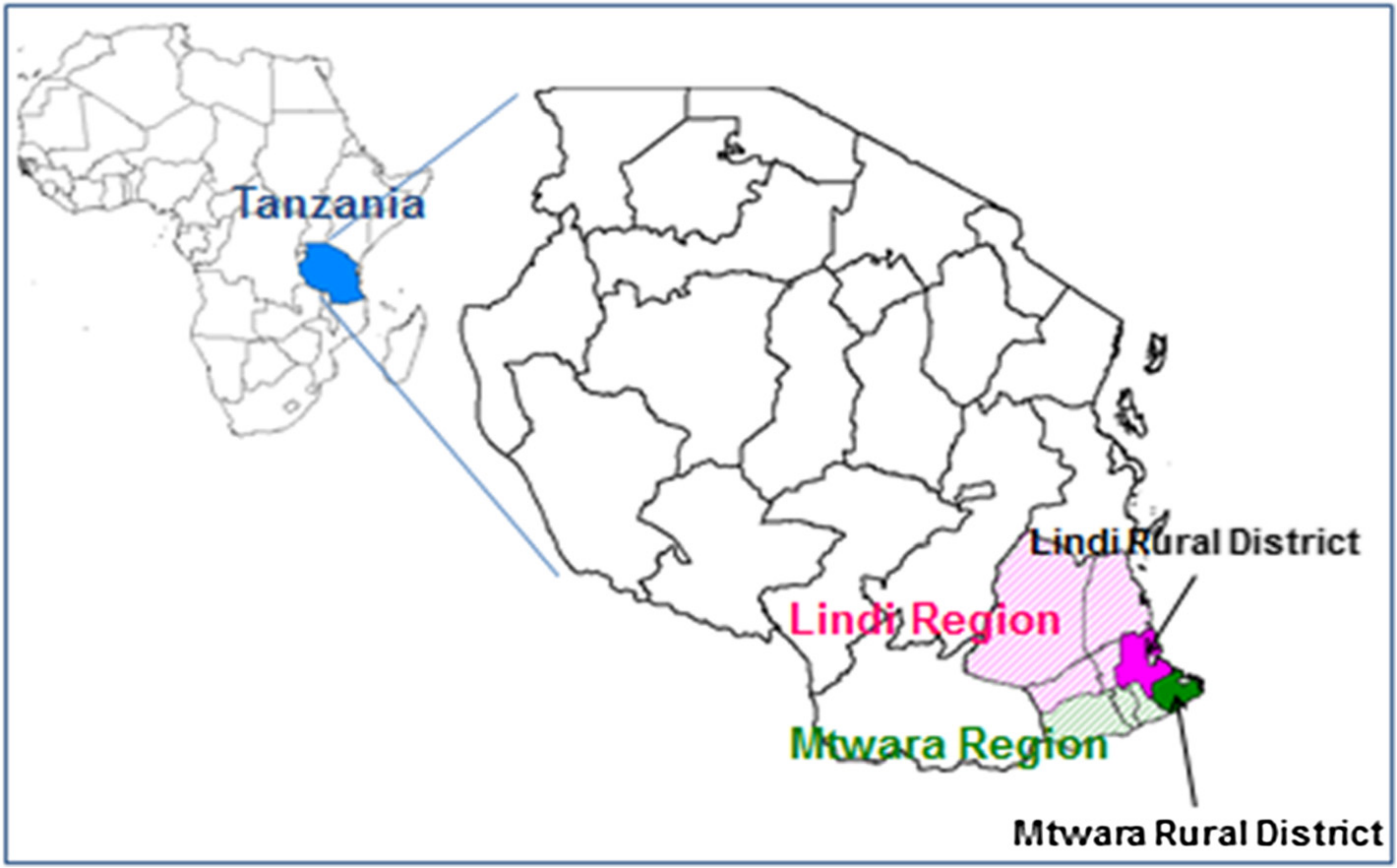
Map of Tanzania showing study districts.

**Figure 2:**
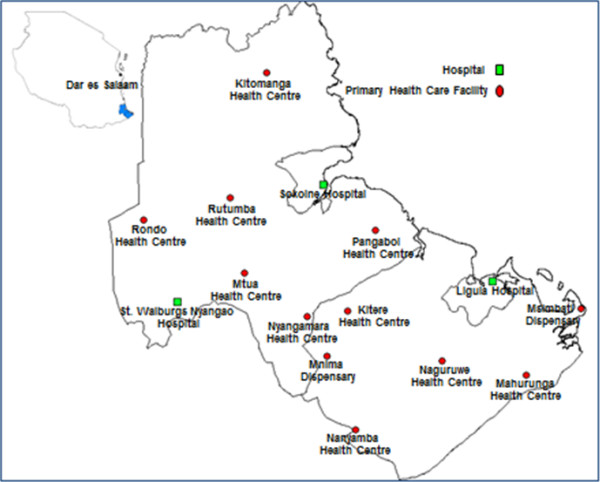
Map of Lindi and Mtwara districts showing study sites.

This study was conducted in eleven primary health care facilities located in two southern regions of Tanzania (Figures 1 and 2), six health facilities in Lindi rural district and five health facilities in Mtwara rural district. Both regions have predominantly agrarian economies. Although the regions have good economic potential in terms of natural resources, their population has incomes around the poverty line and it is here that a large burden of maternal and neonatal morbidity and mortality is found [20].

Lindi region covers 67,000 square kilometers of mainland Tanzania and was projected to have a total population of 905,480 in 2009, the majority (76%) of whom live in rural areas, with an IMR projected at 76 per 1,000 live births [21] and MMR of 132 per 100,000 live births in 2009 [22]. Lindi rural is one of the six districts of Lindi region, covering 7,538 square kilometers, with an estimated population of 244,256 people in 2009. The district has 1 hospital, 5 health centers and 38 dispensaries [20].

Mtwara region, the second smallest region of Tanzania, occupies 16,720 square kilometers of Tanzania mainland area. It is the south-easternmost region of Tanzania, lying in the southern border of Lindi region. Its total population in 2009 was projected to be 1,297,751 and, like its neighbor Lindi, the majority of its people (75%) live in rural areas. MMR was 116 per 100,000 live births in 2009 and IMR was projected to be at 112 per 1,000 live births in 2009 [22,23]. Mtwara region as a whole has 5 hospitals, 170 dispensaries and 19 health centers [20]. Thirty four (34) health facilities, including 4 health centers and 30 dispensaries are in Mtwara rural. Mtwara rural district occupies 3,597 square kilometers of Mtwara region and had 227,134 inhabitants in 2009 [23].

Tanzania has a pyramidal health service structure. Dispensaries and health centers offer the most basic services including MNC (basic obstetric services). Complicated cases are referred to district hospitals or regional hospitals with more specialized maternal services. Ligula Hospital in Mtwara town and Sokoine Hospital in Lindi town offer comprehensive (emergency) obstetric services for Mtwara rural and Lindi rural respectively. Due to some structural challenges, however, the referral system does not work as planned.

### Study design

This was a retrospective quantitative cross-sectional research. Data on costs of health service provision in eleven primary health care facilities for 2009 were collected and analyzed.

This study is part of a research project (Quality of Maternal and Prenatal Care: Bridging the Know-do Gap) funded as part of the 7th Framework Program of the European Union (grant agreement 22982), a collaboration between the Centre de Recherché en Santé de Nouna (Burkina Faso), Ghent University (Belgium), Heidelberg University (Germany), Karolinska Institute (Sweden), Muhimbili University of Health and Allied Sciences (Tanzania), and Navrongo Health Research Centre (Ghana). The selection of the facilities followed the overall project research protocol, where this study is a separate work package.

As part of a baseline study of the multi-country intervention research project to improve quality of MNC, the facilities included in this study were selected as project sites based on two criteria. First, they were typical of the country and second, they were comparable in terms of medical infrastructure, equipment and staffing, corresponding to national standards [24]. The health facilities comprised of one private health center and five public health centers in Lindi rural district and two dispensaries and three health centers, all public, in Mtwara rural district.

A structured questionnaire was developed after a thorough identification of resources likely to be used in the provision of health care services at health centers and dispensaries according to standard national guidelines by the Ministry of Health. Between November and December 2010, data was collected through document reviews, interviews and physical inventory of resources used at the facilities. This enabled the estimation of actual purchased and donated resources used in provision of services in terms of their price and quantity in appropriate units.

Wherever possible, unit prices of resources used were collected directly from health facilities, for example drugs, medical equipment and supplies, personnel (including salaries, benefits, allowances and training) and transport. Some cost data was gathered from the Medical Stores Department (MSD), Expanded Program on Immunization (EPI) and district medical officer’s (DMO) offices. In some cases prices were imputed from market sources, for example prices of some locally obtained equipment including benches, chairs, television set, buckets etc. Building costs were based on estimated replacement cost in these rural areas.

### Cost allocation methodology

Step down cost accounting (SDCA) technique was used to estimate costs. Using this technique a range of resources needed to run a facility were identified and then assigned to chosen cost centers on an allocation basis. The costs in each cost center were aggregated together in overarching themes [25,26]. This methodology was adopted from Conteh and Walker [25] and has been used in various studies [11,14,17,18,27].

The allocation process went through several steps: defining the final product (antenatal and childbirth services), defining cost centers (direct, intermediate and indirect, based on the function of the departments), identifying and grouping the inputs in several categories (personnel, administration, pharmacy etc.), assigning inputs to cost centers reflecting the flow of resources in a health center or dispensary, allocating all costs to final cost centers (reallocation of cost from indirect and intermediate cost centers to direct cost centers (ANC, childbirth)) and ultimately computing total and unit cost for each final cost center.

Our study identified 3 cost centers for analysis. First, indirect cost centers included administration and transport units, these provided general services that were not directly related to final client services but were involved in the overall running of the health facilities. Secondly, intermediate cost centers, pharmacy and laboratory units, incorporated departments that provided diagnostic and departmental support to the final service departments. Finally, direct cost centers included final service departments, ANC, childbirth and other service departments represented the end points of the production line.

Identified resources formed line items for cost categories, (1) Personnel with line items salary, pension, insurance, training, uniform and allowances for all staff, (2) Administration with line items electricity, water, stationery, local transport, communication, cleaning products, repairs and maintenance, (3) Pharmacy with line items drugs and vaccines, (4) Laboratory, (5) Transport with vehicles such as cars, motorbikes and bicycles, (6) Buildings, (7) Medical supplies/consumables and (8) Equipment- medical and office. Full costs incurred by the health facilities were established and used as a control total to ensure that all costs had been distributed to services [25].

The end goal was to allocate all costs to departments that provided direct client care, ANC, childbirth and others. Direct allocation of inputs to cost centers and units was done wherever possible. Some costs can be assigned immediately to certain cost centers. In this case administration, pharmacy, laboratory and transport cost went directly into the respective cost centers and departments. For personnel, buildings, medical supplies and equipment, the average percentage of time spent on activities by staff was the allocation basis.

Staff themselves determined the time they spent on various activities, recording the percentage distribution of their time on a range of activities at the health centers and dispensaries per week. From these reports time estimation was then undertaken for the last twelve months of the study. The health workers recorded the average percentage time spent on categorized health care services over a week’s time period and this was extrapolated to one year. Activities were categorized as administration, vaccine and drug administration, family planning, transportation, ANC, childbirth, post-natal care and “other” activities (“other” captured all other services not included in the mentioned activity categories). From these data, the average time spent on each activity by personnel was calculated per facility and used as the cost allocation basis.

To get the specific cost of providing final services, in our case ANC, childbirth and “other” services, the indirect and intermediate costs were allocated to the direct cost centers using staff time allocation basis. Separate total costs for ANC, childbirth and “other” services were derived.

Service unit costs were then calculated as a ratio of inputs to outputs for ANC and childbirth. Output data reflected service volume (utilization of services), in this case ANC visits and number of normal births in the facilities.

Unit costs reflected productivity in these facilities. Since productivity is one aspect of efficiency, the unit costs therefore reflected efficiency in the provision of MNC services [8,15]. This premise was based on the assumption that, skilled care during pregnancy and childbirth was very likely to reduce maternal mortality, so the number of ANC contacts and the number of professionally assisted childbirths were very good proxy indicators for outcomes and impacts [8]. Efficiency measures how much output a health center or dispensary can produce using a certain amount of input. Therefore those facilities with lower unit costs compared to their counterparts were relatively more efficient in providing ANC and childbirth services.

All costs were identified in local currency, Tanzanian Shillings (TZS), and later converted to United States Dollars (USD) according to the average exchange rate for 2009, 1 USD = 1326.83 TZS. Annuitization was applied to estimate the equivalent annual costs (EAC) for capital outlays [26]. For equipment and vehicles, a discount rate of 3% and useful life of 10 years were used in the calculation. For buildings the same discount rate was used but a useful life of 30 years was considered more appropriate for our context, as applied by Mills et al. [15]. Health care expenditure per capita was calculated as total costs divided by catchment area population. Microsoft Office Excel was used for cost analysis.

### Multivariate analysis

To explain observed unit cost differences between the facilities, a reflection of relative efficiency of providing ANC and childbirth services, we ran a multivariate regression analysis using demand and supply side factors as explanatory variables [13].

Supply/provider side factors included structure and process quality and number of staff. The structure quality reflected the attribute of settings in which MNC services occurred in terms of availability of MNC infrastructure, collected through surveys of the 11 health facilities. The process quality reflected what was actually provided in the giving and receiving of MNC services (quality of care given to women from a health provider) was collected through observations of 418 ANC consultations and 254 childbirths.

Demand/consumer side factors included perceived quality, reflecting the women’s experience of the MNC services provided (women’s satisfaction with care received from a health provider). This was obtained through ANC exit interviews carried out with 611 women, and 503 childbirth exit interviews, and catchment population size obtained through a document review.

The quality variables (structure, process and perceived) indicated various dimensions of quality of MNC services offered in these facilities relative to the World Health Organization (WHO) maternal health services guidelines [28]. Separate quality scores were calculated for antenatal care and childbirth care based on quality measurement scales. Structured checklists were used, if an infrastructure was available and in good working condition, a score of ‘1’ was given for the health facility survey, for the observation study a score of ‘1’ was given if the activity was observed and performed according to accepted standards of care and a score of ‘0’ was given otherwise. A 5-point Likert scale with scores ranging from ‘+2’ meaning ‘very satisfied’ to ‘-2’ meaning ‘very unsatisfied’ was used for the satisfaction survey and factor-analysis was performed. The demand and supply side data were collected as part of a baseline quality assessment of MNC services in the health facilities [24]. Details around data collection and analysis of the quality scores can be found elsewhere [29].

We used SPSS version 16 to perform a stepwise backward elimination process in model estimation. The method started with all independent variables (Equation 1) entered in a linear regression, and then step-by- step variables with smallest *t statistic* and *p-value* of at least 0.100 were removed to get the most significant model. We tested several models but reported only the most significant ones.

(1) $$\mathrm{Unit}\;\cos t_{\mathrm{ANC}\;\mathrm{or}\;\mathrm{childbirth}}=f\;\left(\begin{array}{c} \mathrm{process}\;\mathrm{quality}\;\mathrm{of}\;\mathrm{care},\mathrm{structural}\\ \mathrm{quality}\;\mathrm{of}\;\mathrm{care},\;\mathrm{perceived}\;\mathrm{quality}\;\mathrm{of}\\ \mathrm{care},\;\mathrm{number}\;\mathrm{of}\;\mathrm{staff},\mathrm{catchment}\\ \mathrm{population}\;\mathrm{and}\;\mathrm{population}\;\mathrm{staff}\;\mathrm{ratio} \end{array}\;\right)$$

## Results

### Activity time allocation

Reported average time spent per activity by staff in a health center or dispensary was the allocation basis for cost. This allowed us to gain a clear picture of the share of each resource used in the provision of health care services, the average percentage time spent on an activity per health center or dispensary and for the two districts as a whole. On average, about 8% (range 3-15%) and 10% (range 4-20%) of staff time was spent on provision of ANC and childbirth services respectively. The rest of time was divided between the other activities (Table 1).

**Table 1:** Personnel reported time allocation by health facility and activity in percentage, 2009

**Health facility**	**Administration**	**Transport**	**Pharmacy and laboratory**	**ANC**	**Childbirth**	**Other services**	**Total time %**
**Mtwara Rural District**							
HF 1 HC	8	11	24	6	8	43	100
HF 2 HC	11	9	25	6	7	42	100
HF 3 Dis	13	0	30	7	10	40	100
HF 4 Dis	15	0	30	5	20	30	100
HF 5 HC	11	0	30	9	9	41	100
**Lindi Rural District**							
HF 1 HC	19	0	30	3	13	35	100
HF 2 HC	7	14	21	7	4	47	100
HF 3 HC	7	0	30	13	17	33	100
HF 4 HC	8	0	30	10	11	41	100
HF 5 HC	9	0	30	15	10	36	100
HF 6 HC	14	0	26	10	6	44	100
**Average %**	**11.09**	**3.09**	**27.82**	**8.27**	**10.45**	**39.27**	**100**

HF = Health Facility.

HC = Health Center.

Dis = Dispensary.

Figures rounded to nearest whole numbers.

### Cost composition, distribution and determinants

For all the surveyed health facilities in both rural districts, the total running cost in 2009 was approximately 623,908 USD (Table 2). Mtwara rural district consumed around 271,159 USD while Lindi rural district consumed around 352,750 USD. In Mtwara rural district, the average total cost of providing services was approximately 54,232 USD with a wide range of 21,279 USD to 84,866 USD while in rural Lindi it was 58,792 USD, with a range 43,660 USD to 68,475 USD.

**Table 2:** Cost, service volume, catchment population and number of health workers by health facility, 2009

**Health facility**	**Cost per ANC visit USD**	**Cost per childbirth USD**	**Number of ANC visits**	**Number of births**	**Catchment area population**	**Number of staff**	**Total cost**
**Mtwara Rural District**							
HF 1 HC	5.55	80.57	883	83	9325	7	65808.78
HF 2 HC	8.05	38.47	920	233	10978	9	84865.78
HF 3 Dis	6.63	40.10	392	95	6010	3	31522.90
HF 4 Dis	2.87	61.74	468	87	4878	2	21279.23
HF 5 HC	12.79	53.47	581	139	6312	8	67682.09
**Mtwara total**			**3244**	**637**		**29**	**271158.8**
**Mtwara average**	**7.18**	**54.87**					**54231.76**
**Lindi Rural District**							
HF 1 HC	10.88	139.62	138	50	10310	6	43660.32
HF 2 HC	22.47	32.68	188	106	3551	5	57216.61
HF 3 HC	59.48	211.53	145	56	14526	3	55812.04
HF 4 HC	14.79	51.77	548	170	6663	6	64944.41
HF 5 HC	30.24	122.97	374	63	5832	4	62641.61
HF 6 HC	6.85	44.70	1325	123	10624	6	68474.52
**Lindi total**			**2718**	**568**		**30**	**352749.5**
**Lindi average**	**24.12**	**100.55**					**58791.59**
**Overall total**			**5962**	**1205**	**89009**	**59**	**623908.3**
**Overall average**	**16.42**	**79.78**					

HF = Health Facility.

HC = Health Center.

Dis = Dispensary.

Unit costs varied widely between the facilities. On average, unit cost for ANC was 16.42 USD, with a wide range of 2.87 USD to 59.48 USD. Number of ANC contacts for both districts totaled 5,962 visits, with a wide range from 138 contacts to 1,325 contacts per facility (Table 2). Childbirth unit cost was on average 79.78 USD with a wide range of 32.68 USD to 211.53 USD. The number of births ranged from 50 to 233 births, both districts had 1,205 births in total (Table 2).

Overall personnel consumed the biggest part of resources at approximately 44% (Table 3). Allocation of total running costs to the final services showed that approximately 11% of total cost in both rural districts went to ANC and 12% went to childbirth services. Around 12% and 13% of Lindi total resources went to the provision of ANC and childbirth services while in Mtwara it was 9% and 12%. Approximately 7 USD was spent on providing health care services per capita, 7.23 USD and 6.84 USD for Lindi and Mtwara respectively.

**Table 3:** Percentage cost distribution in all facilities by district, 2009

**Cost item**	**Percent distribution Mtwara**	**Percent distribution Lindi**	**Percent distribution all facilities**
Personnel	51	38	44
Administrative	4	22	15
Pharmacy and medical supplies	19	12	15
Transport	4	8	6
Buildings	13	15	14
Equipment	9	5	6
**Total %**	**100**	**100**	**100**

Regression analysis showed that, changes in factors like women’s perceived quality of care, structural quality of care, process quality of care and number of staff may have led to changes in ANC unit costs (efficiency) and were significant (p = 0.044, p = 0.000, p = 0.003 and p = 0.002 respectively) predictors of ANC unit costs in these facilities (Table 4). Both number of staff and process quality had negative influence on unit costs. On the other hand, structural quality (infrastructure availability) and women’s perceived quality of ANC care had positive effect on unit costs.

**Table 4:** Multiple regression results, using stepwise backward elimination method

**Variable**	**Description**	**B coefficient**	**SE**	**t**	**P**
Number of staff	Continuous variable	-4.577	0.85	-5.385	0.002
Structure quality ANC	Score 0-1	201.182	22.849	8.805	0.000
Process quality ANC	Score 0-1	-205.848	43.799	-4.7	0.003
Women’s perceived quality of ANC care	Dummy variable, satisfied = 1	11.285	4.455	2.533	0.044

Dependent variable: Cost per ANC visit in USD.

F statistic = 26.254.

p of F statistic = 0.001.

R-square = 0.946.

Adjusted R-square = 0.910.

Catchment area population size was not correlated with cost per childbirth (Table 5). Structural quality (without considering availability of infrastructure for basic emergency obstetric care (BEmOC) services) and process quality of childbirth had very marginal influence on cost per childbirth. Increase in structural and process quality were correlated with lower unit costs (p = 0.08 and p = 0.07). On the other hand, population-staff ratio and availability of infrastructure for basic emergency obstetric care (BEmOC) services had significant positive influence on unit costs (p = 0.022 and p = 0.029).

**Table 5:** Multiple regression results, using stepwise backward elimination method

**Variable**	**Description**	**B coefficient**	**SE**	**t**	**P**
Catchment population	Continuous variable	0.007	0.004	1.71	0.148
Population-staff ratio	Catchment population/number of staff	0.03	0.009	3.281	0.022
Structure quality	Score 0-1	-280.905	128.049	-2.194	0.08
BEmOC services availability	Score 0-1	171.258	56.594	3.026	0.029
Process quality	Score 0-1	-118.107	51.316	-2.302	0.07

Dependent variable: Cost per childbirth in USD.

F statistic = 9.905.

p of F statistic = 0.012.

R-square = 0.908.

Adjusted R-square = 0.817.

## Discussion

This paper reported the actual use of financial resources in the provision of health services with a focus on maternal and newborn care (ANC and childbirth) in 11 rural settings of Tanzania. It gave a clear picture of costs and their distribution. Further, it shows factors that influenced differences in ANC and childbirth unit costs between the health facilities taking into account perceived, structure and process quality, number of staff and catchment area population size. Efficiency in the provision of ANC and childbirth services was reflected by the unit costs.

### Health care resource use

This study estimated that approximately 7 USD was spent to provide health care per person in both districts giving a sense of resource distribution equality between the rural districts. This figure nevertheless reflected resource scarcity for health services. The expenditure was roughly 26% of the national health expenditure per capital in 2009 [7]. Furthermore, this figure is below the WHO standard, where on average a low-income country has to spend a minimum of 44 USD per capita [1] to ensure all people have access to a set of essential health services including maternal and child health. Dispensaries had the smallest amount of resources directed to them. This is because Tanzanian government allocates resources to health facilities according to their level of service provision.

Personnel costs accounted for the largest portion of total costs in these facilities. Mtwara had a slightly higher proportion than its neighbor Lindi due to variations in staff qualifications, performance based incentives and allowances observed in this study. Similar studies [8-11] have also found health care to be a labor-intensive sector. Drugs and medical supplies and administration made the second largest consumption groups followed by buildings, transport and equipment (these three represented the capital costs) for the two districts. Past studies have also found drugs and medical supplies to make the second largest cost component in health facilities [8,9]. Administrative costs were noticeably higher in Lindi compared to Mtwara due to higher electricity and other operational costs. It is crucial that these limited resources are used efficiently to ensure that the population fully benefit from them.

### ANC and childbirth service utilization and unit costs

Our study showed a low utilization of ANC and childbirth services and a wide variation in unit costs between the facilities. Low utilization implies that the direct costs have to be spread across fewer units of output as reflected by the unit costs [16]. Most women in Tanzania book late for ANC and have less than the four visits per pregnancy that is recommended by the WHO and the Tanzania Ministry of Health [3,30,31]. Just 15% of women have undergone an ANC visit by their fourth month of pregnancy [31]. Home births are more common in rural areas (56%) than in urban areas. Overall, only about half of Tanzania’s births occur in health facilities [31,32]. Apart from these reasons, one other substantial limitation factor is the trade-off between the access to health facilities and price of health services faced by women. This has an important implication on the argument for dimensions of improvement of efficiency, especially in remote areas with limited resources.

### Efficiency- quality relationship

Availability of health care infrastructure has been found to have positive effect on utilization [33,34]. The study facilities had most infrastructures for MNC provision in place. However, none of the health facilities had the required infrastructure for providing basic emergency obstetric care (BEmOC) services. Investment in BEmOC has a potential to impact positively the utilization and efficiency of services. A stronger emphasis should be placed on improving the current facilities, to enable provision of basic emergency obstetric care, and efficient use of the existing facilities. Improvement in process quality is also vital for efficiency.

How women perceive quality of care affects their utilization levels [31,34,35]. Some women bypass nearby health facilities and spend more time and money to deliver at health facilities where the quality is, or is perceived to be higher [8,33,34,36]. Our study suggests that an improvement in ANC process quality will appeal to women in terms of their perception and thus they will utilize the services offered. Our study has shown ANC perceived quality to be positively influenced by ANC process quality (p = 0.026). Therefore given the availability of infrastructure for ANC, these facilities should focus on process quality improvement with a view of efficiency gains.

Unlike for ANC, a woman’s perception of quality of care had no association with childbirth unit costs. Given the emergency nature of childbirth a woman’s perceived quality of care may not really play a strong role on the final decision on where to deliver. Furthermore, rural families base their decisions to utilize health care providers for childbirth, including emergency births, not only on quality criterion but also on a number of other criteria including out of pocket cost, opportunity cost, transport access and how the community perceives a woman’s health condition among other things [17,32,33,36-43]. Usually the husband/partner and close relatives are actively involved in deciding where a woman gives birth [37].

Many other studies have shown the importance of quality of care for utilization of professional assistance during pregnancy and childbirth, including the importance of availability of health workers [34-39,44]. There is a scarcity of health care personnel especially in remote rural areas of Tanzania, discouraging utilization. Normally the available few providers are required to stretch to a number of services including MNC. Our study suggests an investment in staff to cater for clients may lead to efficiency gains especially for ANC. This will mean more time per woman and reduced waiting time for the services resulting to increased utilization, reduction of unit costs and improved efficiency.

Our study suggested that, improvement in quality dimensions involving number of health workers and how they provide services to women had a positive effect on efficiency however, trade-offs between quality and efficiency are inevitable especially when making investments. Costs are incurred when investments are made to improve health service provision; a case in point was the importance of investing in BEmOC regardless of the cost involved because none of the facilities under study met the criteria for proper provision of this crucial maternal service.

### Staff time allocation

Our study showed a wide range of time allotted to ANC and childbirth services between study facilities. Besides differences in service volume as one explanation, this study does not have more evidence to explain this variation. However, past studies have found that Tanzanian health facilities do not have an official structure for service delivery, especially for preventive services. Health workers in some facilities tend to overcrowd clients in the morning hours leaving the afternoon hours almost unoccupied [8,12,45].

### Limitation of the study

Due to the small number of health facilities involved in this study, representativeness of the results cannot be ascertained. These results are applicable to the study districts. For countrywide health care policy-making and planning larger samples are required. The retrospective methodology used is also a limitation. A prospective approach would have been useful in capturing actual time allocation for health services by providers in addition to the recall approach used. A qualitative investigation on the acceptance and preferences of the women for explanation of the level of utilization of services would have made our study findings more robust. This would have also addressed the possible effects of unit cost from the perspective of district and facility managers and health providers.

Comparing our study to a similar study conducted in Mtwara urban health facilities by Both et al. [8] shows that this study’s unit costs are higher. Our results are comparable to a review study by Borghi [46] for developing countries, in which cost per ANC ranged from 2.21 to 42.41 and for normal vaginal childbirth, ranged from 2.71 to 140.41. The main reasons for these observed variations are the differences in service volume, input costs and methodology. Both et al. [8] used randomized intermittent instantaneous observations in addition to recall in estimating time allocation but did not account for all the costs due to data unavailability at the time of their study. Furthermore, Both et al. [8] reported higher number of ANC visits and childbirths per year.

## Conclusions

Our study has shown great variability in unit costs of ANC and childbirth service provision, reflecting room for efficiency improvement. High unit costs are mainly due to level of input costs and service utilization in these facilities. The importance of focusing on providers and consumers of health care in efforts to improve efficiency cannot be over emphasized.

With our study sites having most infrastructures for provision of ANC and childbirth services, a focus should be on improving process quality and putting in place infrastructure for basic emergency obstetric care services. Restructuring of the referral system to ensure effective response to obstetric and newborn emergencies is also key.

Improvement of human resources in terms of number and competency is also important. Human resources make most part of costs therefore this warrants a careful analysis of health facilities, including analysis of the health care provision structure, to identify areas with human resource improvement need.

Fixed costs (buildings, equipment and personnel) make the largest share of total costs, showing opportunity for efficiency improvement. In those facilities operating below full capacity, unit costs are likely to fall with increment in MNC utilization, at least in the short run as most costs are fixed [47]. Reduction of resource waste through increased utilization of MNC services can improve efficiency of service delivery in those areas operating below capacity [13,19,27,48].

Health care demanders are heterogeneous and they incorporate a range of criteria in their decision to utilize ANC and childbirth services. Apart from perceived quality of care, unofficial charges, opportunity costs, transport accessibility plus a whole range of other issues are important for utilization. Keeping this in mind, focused maternal interventions are envisioned to work in increasing utilization compared to generalized interventions [38]. Community participation has been shown to be effective [49]. Therefore, the district health management should find ways in which the community can be involved to ensure women’s use of these essential MNC services.

The results of this study are important for sensitization of district management towards performing and standardizing cost assessment in different health facilities in Tanzania. The routine performance of such analyses would reduce the cost for the analyses compared to the benefits expected. Efficiency analyses through simple techniques such as measurement of unit costs should be made standard in health care provision, health managers can then use the performance results to gauge progress and reward efficiency through performance based incentives. In addition, the results are important to inform the health policy on the possibilities for higher utilization of MNC services through improvements in process quality, human resources structure and involving the community.

## Abbreviations

ANC: Antenatal care; BEmOC: Basic emergency obstetric care; DMO: District medical officer; EPI: Expanded program on immunization; IMR: Infant mortality rate; MNC: Maternal and newborn care; SDCA: Step-down cost accounting; URT: United Republic of Tanzania; USD: United States Dollar; WHO: World Health Organization.

## Competing interests

The authors declare that they have no competing interests.

## Authors’ contributions

HPS acquired analyzed and interpreted data and drafted the manuscript. ED HD SM RS and SL critically revised the content. PT assisted in regression analysis. MD and GS assisted in cost data analysis and review of content. All authors read and approved the final manuscript.

## Pre-publication history

The pre-publication history for this paper can be accessed here:

http://www.biomedcentral.com/1472-6963/14/96/prepub
